# Stress‐Regulation Design of Mesoporous Carbon Spheres Anodes with Radial Pore Channels Toward Ultrastable Potassium‐Ion Batteries

**DOI:** 10.1002/smsc.202200045

**Published:** 2022-09-20

**Authors:** Shuming Dou, Qiang Tian, Tao Liu, Jie Xu, Lingyan Jing, Cuihua Zeng, Qunhui Yuan, Yunhua Xu, Zheng Jia, Qiong Cai, Wei-Di Liu, S. Ravi P. Silva, Yanan Chen, Jian Liu

**Affiliations:** ^1^ School of Materials Science and Engineering Key Laboratory of Advanced Ceramics and Machining Technology of Ministry of Education Tianjin Key Laboratory of Composite and Functional Materials Tianjin University Tianjin 300072 China; ^2^ School of Materials Science and Engineering Harbin Institute of Technology (Shenzhen) Shenzhen 518055 China; ^3^ Dalian National Laboratory for Clean Energy State Key Laboratory of Catalysis Dalian Institute of Chemical Physics, Chinese Academy of Sciences Dalian 116023 China; ^4^ Key Laboratory of Soft Machines and Smart Devices of Zhejiang Province Center for X-Mechanics, Department of Engineering Mechanics Zhejiang University Hangzhou 310027 China; ^5^ DICP-Surrey Joint Centre for Future Materials Department of Chemical and Process Engineering Advanced Technology Institute University of Surrey Guilford Surrey GU2 7XH UK; ^6^ Australian Institute for Bioengineering and Nanotechnology The University of Queensland St Lucia Brisbane Queensland 4072 Australia

**Keywords:** finite element simulations, mesoporous carbon spheres, potassium-ion batteries, radial pore channels, stress-buffering effect

## Abstract

Electrochemical energy storage (EES) devices are expected to play a critical role in achieving the global target of “carbon neutrality” within the next two decades. Potassium‐ion batteries (KIBs), with the advantages of low cost and high operating voltage, and they could become a major component of the required energy‐material ecosystems. Carbon‐based materials have shown promising properties as anode materials for KIBs. However, the key limitation of carbon anodes lies in the dramatic mechanical stress originating from large volume fluctuation during the (de)potassiation processes, which further results in electrode pulverization and rapid fading of cycling performance. Here, a controllable self‐assembly strategy to synthesize uniform dual‐heteroatom doped mesoporous carbon sphere (DMCS) anodes with unique radial pore channels is reported. This approach features a modified Stöber method combined with the single‐micelle template from the molecule‐level precursor design. The DMCS anodes demonstrate exceptional rate capability and ultrahigh cycling stability with no obvious degradation over 12 000 cycles at 2 A g^−1^, which is one of the most stable anodes. Furthermore, finite element simulations quantitatively verify the stress‐buffering effect of the DMCS anodes. This work provides a strategy from the perspective of stress evolution regulation for buffering mechanical stress originating from large volume fluctuations in advanced KIBs electrodes.

## Graphical abstract

Outstanding performance in ultralong cycling stability is shown for nanostructured carbon‐based anodes. In this work, finite element simulations are combined to study the dual‐heteroatom doped mesoporous carbon spheres (DMCSs) to reveal the origin of their ultrastable potassium‐ion storage.

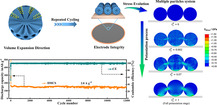

## 1 Introduction

With the increasingly severe energy and environmental crisis faced by the world with regard to carbon emission, carbon neutrality has become a global focus.^[^ ^1^ ^]^ Electrochemical energy storage (EES) technologies, particularly rechargeable secondary batteries, offer a promising and sustainable route to achieving carbon neutrality.^[^ ^2^ ^]^ Over the past decades, researchers have witnessed the successful commercialization of lithium‐ion batteries (LIBs) in a wide range of applications, ranging from portable devices to hybrid/full electric vehicles.^[^ ^3^ ^]^ However, the limited lithium resources in the earth's crust (0.0017 wt%) and the growing demand for power grid storage have triggered numerous efforts to develop next‐generation EES devices beyond the LIBs.^[^ ^4^ ^]^ Recently, potassium‐ion batteries (KIBs) have received considerable attention due to the natural abundance and cost‐effectiveness of *K* resources (1.5 wt%), and its suitable redox potentials (–2.93 V vs standard hydrogen electrode, SHE) close to Li (–3.04 V vs SHE).^[^ ^5^ ^]^ A pivotal prerequisite to achieving high‐performance KIBs lies in designing high‐capacity anode materials, which is mainly limited by the large volume variation and sluggish kinetics deriving from the larger ionic radius of *K* (1.38 Å) compared to that of Li (0.68 Å).^[^ ^6^ ^]^ Correspondingly, various types of anode materials for KIBs (i.e., alloying, intercalation, conversion) such as metal/alloy,^[^ ^7^ ^]^ carbon‐based materials,^[^ ^8^ ^]^ transition‐metal oxides/sulfides/selenides^[^ ^9^ ^]^ have been investigated. Carbonaceous materials hold promising properties for commercial application due to their abundant storage, eco‐friendly peculiarity, and structure controllability.^[^ ^10^ ^]^ However, carbonaceous electrode materials undergo dramatic mechanical stress originating from huge volume fluctuation during insertion/extraction of *K* ions, which leads to pulverization of the electrode, loss of the electrical contact, and consequently rapid fading of storage performance.^[^ ^11^ ^]^

Various strategies have been attempted to effectively buffer the stress‐driven degradation of carbon‐based anodes. Porous structural engineering is an effective strategy to alleviate the huge volume strain of carbonaceous materials.^[^ ^12^ ^]^ The incorporation of a highly developed porous structure can provide sufficient elastic buffering space to alleviate the volume fluctuation during potassiation. Nevertheless, the formation of pores in carbon is usually accompanied by using redundant sacrificial agents (e.g., KOH and HF),^[^ ^13^ ^]^ resulting in the complexity of the synthetic process. The chemical etching method is difficult to accurately control the size and structure of the resultant nanopores. Besides, the construction of hollow micro/nanostructures can accommodate the mechanical strain caused by consecutive ion insertion/extraction process, increase the contact between the anode and electrolyte, and realize superior cycling stability.^[^ ^14^ ^]^ Recently, a hollow carbon nanosphere with a single‐shelled configuration has been synthesized by various methods, including chemical etching,^[13a]^ chemical vapor deposition,^[^ ^15^ ^]^ and thermal decomposition,^[^ ^16^ ^]^ which can effectively release the radial stress of the single‐shelled hollow structure. However, it still suffers from high stress in the tangential direction originating from the inevitable inhomogeneous electrode reaction,^[^ ^17^ ^]^ where the fabricated electrodes can be destroyed in multiple particle systems. Therefore, increasing the diversity of pore structures in carbon spheres and preparing hollow carbon nanospheres with porous architectures is vitally important for alleviation of volume strain in the practical electrodes.

Stress management of anode materials during (de)potassiation is critical to design rational micro/nanoarchitectures of electrode materials aiming at mechanical stress relaxation and cycling performance improvement.^[^ ^18^ ^]^ Nanoengineering is significantly effective in releasing the high mechanical stress of the electrodes. However, the intercorrelations between the nanostructured configuration of the active materials, the mechanical stress distribution, and the electrochemical *K* storage performance have been rarely studied. Therefore, the research of internal stress evolution in the electrochemical reaction process is of great importance. In terms of single‐carbon‐particle‐based systems, maintaining the structural integrity of electrodes is the essential prerequisite for approaching remarkable electrochemical properties. Hence, an accurate understanding of the stress evolution during (de)potassiation is of great significance to smart anode material design. However, the related studies are still insufficient, where the structural design of carbonaceous materials is rather intuitive. More importantly, from a practical application viewpoint, the stress evolution of multiple‐carbon‐particle‐based systems differs from that of single‐carbon‐particle‐based systems.^[^ ^19^ ^]^ The stress evolution of multiple particle electrodes is significantly affected by the interaction between carbon‐based particles. Therefore, it is important to consider the stress evolution between different particles in multiple particle systems during (de)potassiation to achieve ideal functional architectures with strong mechanical structure stability and satisfying cycling performance.

**Figure 1:**
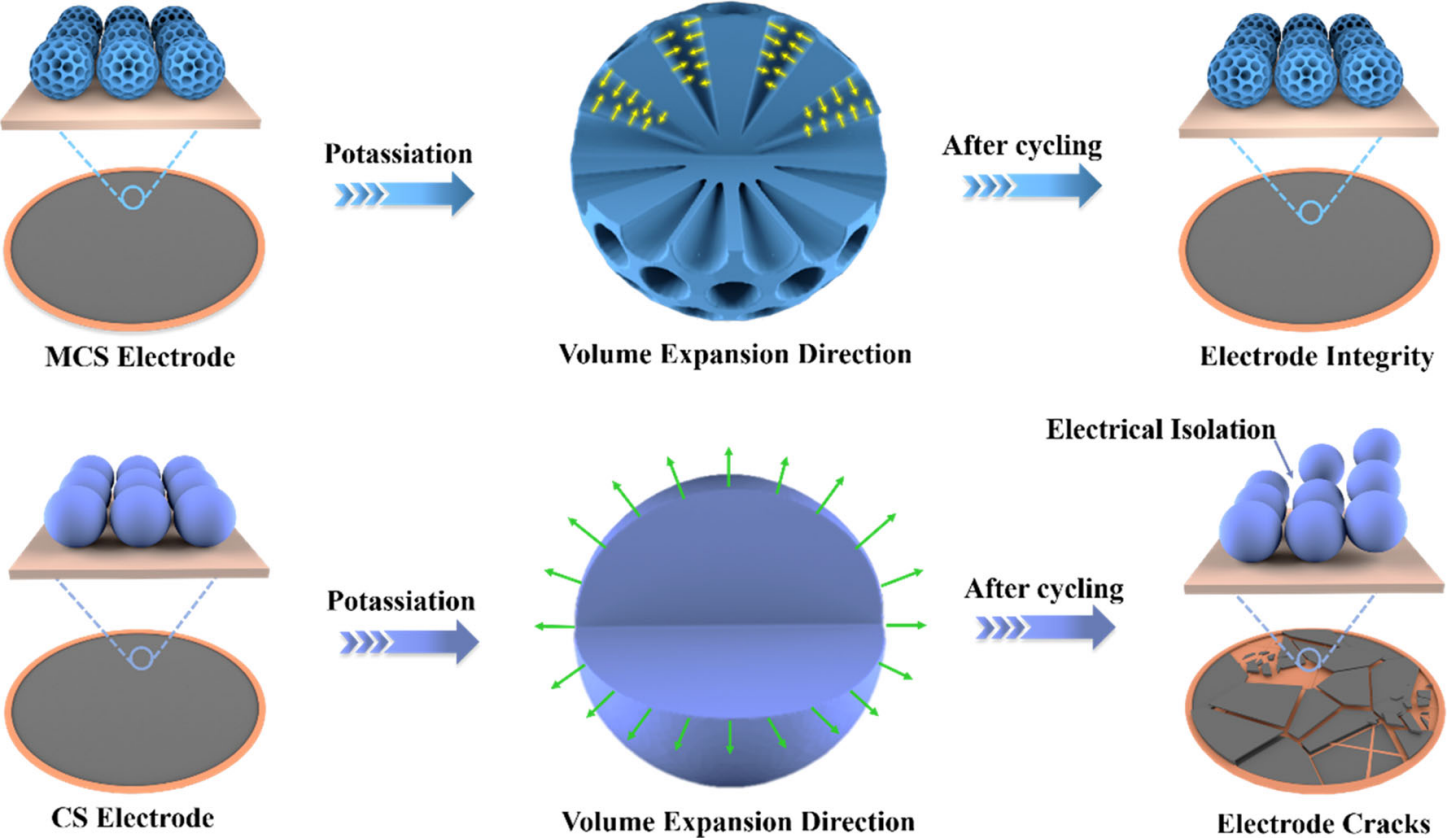
Schematic diagrams of the stress evolution management in non‐mesoporous carbon sphere (CS) and mesoporous carbon sphere (MCS) electrodes.

Here, we report a modified Stöber method combined with the single‐micelle template approach to fabricate uniform dual‐heteroatom doped mesoporous carbon spheres (DMCSs) with unique radial pore channels from the molecule‐level precursor design. Such a unique nanoengineering approach renders multiple conspicuous merits for huge‐volume‐fluctuation carbon‐based anode materials. The DMCS anode delivers outstanding rate capability and ultralong cycling stability over 12 000 cycles at 2 A g^−1^. To gain an insight into associations between the nanoengineering design and the ultrastable potassium‐ion storage, mechanical simulation is applied to model the von Mises stress distribution of two representative structures of carbon electrode particles (non‐mesoporous carbon sphere (CS) and mesoporous carbon spheres (MCS)) in the single/multiple particle systems under an isotropic initial stress by the finite element method. The simulation results quantitatively manifested that MCS can facilitate the reduction of stress associated with volume variation during the charge/discharge process compared with CS. Therefore, the DMCS with unique radial pore channels can contribute to the structural integrity of the electrodes and the exhibition of superior long‐term cyclic stability (**Figure** **1**). This work renders a rational design and stress evolution regulation of anode materials for advanced KIBs.

## 2 Results and Discussion

**Figure 2:**
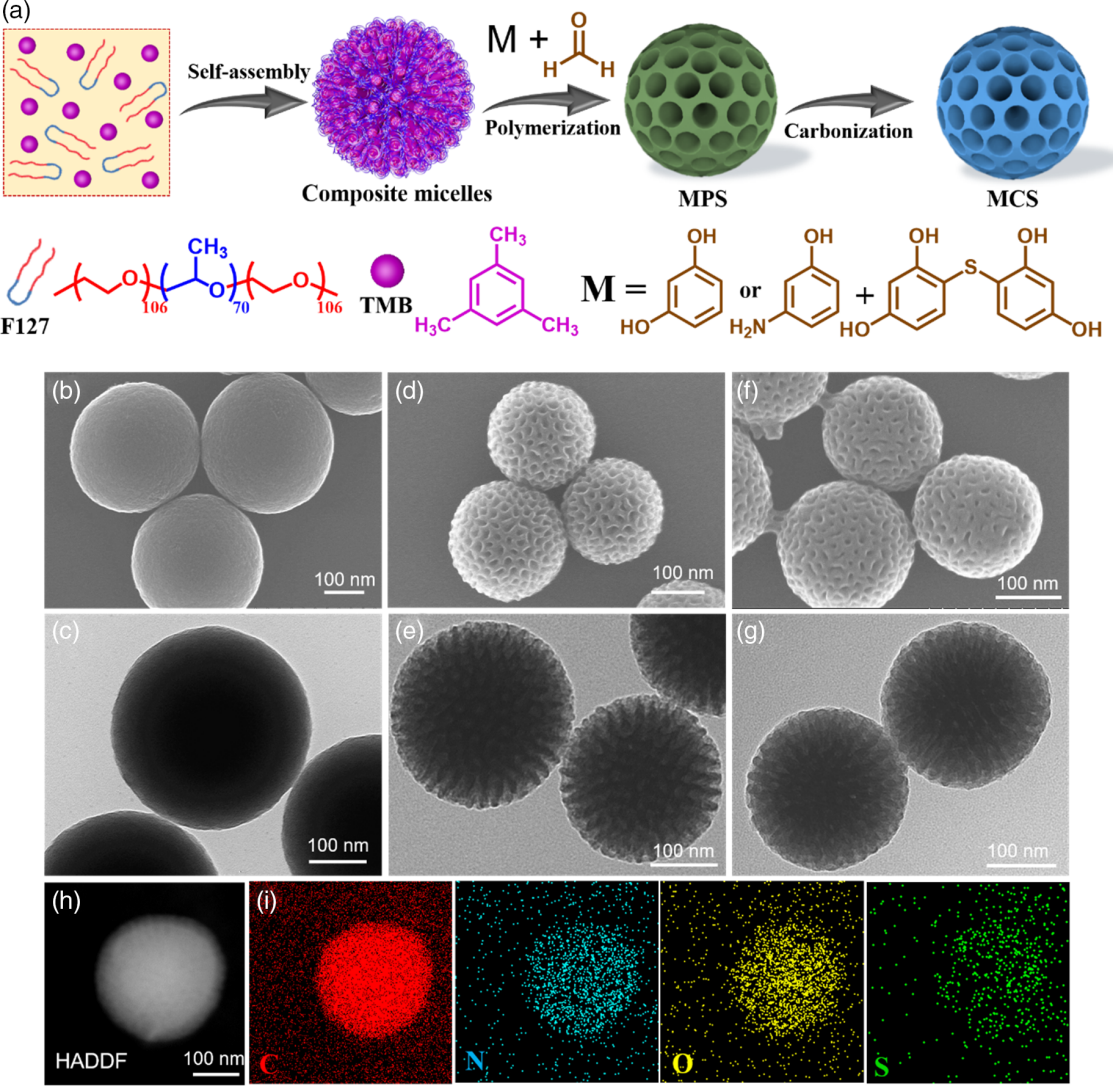
a) Schematic representation of the general method for synthesizing hetero‐element doped porous carbon spheres. b–g) SEM and TEM images of the samples: b,c) CS, d,e) MCS, and f,g) DMCS. h) High‐resolution TEM (HRTEM) image and i) EDS mapping of DMCS.

The extended Stöber method was modified and combined with the single‐micelle template approach to fabricate hetero‐element‐doped mesoporous carbon spheres with unique radial pore channels from the molecule‐level precursor design (**Figure** **2**a).^[^ ^20^ ^]^ The resorcinol‐formaldehyde precursors show similar coordination sites and tetrahedral geometry compared with silanes.^[^ ^21^ ^]^ The triblock copolymer F127 (PEO_106_PPO_70_PEO_106_) was employed as a single‐micelle template.^[^ ^22^ ^]^ The 1,3,5‐trimethylbenzene (TMB) was employed as a mediator and swelling agent in the ethanol/water system to synthesize monodisperse carbon spheres (CS).^[^ ^23^ ^]^ TMB can enter the poly(propylene oxide) (PPO) core of F127 to swell the micelle.^[^ ^24^ ^]^ Following the polymerization of resorcinol and formaldehyde onto the poly(ethylene oxide) (PEO) domains of F127, mesoporous resorcinol–formaldehyde polymeric materials are generated under the catalysis of ammonia.[^24^, ^25^] Because the colloidal spheres have low surface energy,^[^ ^26^ ^]^ the finally synthesized material has a mesoporous RF sphere, which can then be carbonized into mesoporous carbon spheres (MCS). In addition, different phenolic precursors are suitable for the extended Stöber method and single‐micelle template. 3‐aminophenols with amino groups (—NH_2_) and resorcinol sulfide with thioether bonds (—S—) can replace resorcinol for the design of nitrogen/sulfur dual‐doped mesoporous carbon spheres (DMCS) from the molecule level. Thereby, after carbonization, DMCS can be synthesized.

The refined morphology of the products (CS, MCS, DMCS) was revealed by electron microscopy characterization. As shown in the scanning electron microscopy (SEM) images (Figure S1c, Supporting Information), the DMCS shows a uniform spherical morphology in the size of about 200 nm. Close observation of the DMCS reveals the existence of homogeneously distributed dense holes on the surface of each nanoparticle (Figure 2f). The transmission electron microscopy (TEM) image (Figure 2g) clearly demonstrates that the meso‐channels are arranged radially from the center to the surface. Interestingly, the morphology and internal microstructure of the MCS are similar to those of the DMCS, as shown in Figure 2d,e and S1b, Supporting Information. With the shearing flow caused by the agitation, the large spherical F127/TMB micelles are impacted, deformed, and fused along the radial direction, which correspondingly leads to the self‐assembly of polymer oligomers into radially orientated mesoporous spheres.^[23c]^ The CS has a smooth surface and displays no internal channel configuration as seen in the TEM and SEM images (Figure 2b,c and S1a, Supporting Information). The high‐angle annular dark‐field scanning transmission electron microscopy and energy‐dispersive X‐ray spectroscopy (EDS) elemental maps of the DMCS in Figure 2h,i show a uniform elemental dispersion of C, N, O, and S over the carbonaceous matrix. The Brunauer–Emmett–Teller (BET) specific surface area and the corresponding pore size distribution of these three samples were evaluated by the N_2_ adsorption/desorption analyses as shown in Figure S2a–c, Supporting Information. The DMCS possesses a high BET‐specific surface area of 426.39 m^2^ g^−1^, an average pore diameter of 7.89 nm, and a total pore volume of 0.39 cm^3^ g^−1^, which is in line with the morphology observed from the SEM and TEM characterizations. As depicted in Figure S2b,c, Supporting Information, the DMCS has a mesopore‐dominated pore size distribution in a broad range of 2.60–88.61 nm. The surface area and pore texture of the MCS is similar to those of the DMCS. In contrast, the pore size distribution of CS is indicative of the absence of mesoporous architecture. Hence, both MCS and DMCS present the channel‐structured pores, suggesting distinct intrinsic mesoporosity. The abundant pore structure provides channels for the rapid transportation of ion diffusion, and potential stress‐buffering space for volume expansion, contributing to the exceptionally enhanced rate performance and cycling stability observed.^[^ ^27^ ^]^

The two broad peaks corresponding to (002) and (100) diffractions in the XRD pattern of the DMCS (Figure S2d, Supporting Information) signify the low graphitization degree. As a supplement, the Raman spectrum (Figure S2e, Supporting Information) depicts two peaks near 1339 and 1588 cm^−1^, which are indexed to the defect/disorder‐induced D‐band and in‐plane C–C bonds vibrational G‐band, respectively.^[^ ^28^ ^]^ The *I*_D_/*I*_G_ intensity ratios of 0.963, 0.963, and 0.975 for CS, MCS, and DMCS, respectively, are calculated from the Raman spectrum, indicating the considerably defective structure and the low crystallinity degree in the carbon matrix. The aforementioned results show that MCNS exhibits a typical amorphous carbon structure. According to the X‐ray photoelectron spectroscopy (XPS) analysis (Figure S2f, Supporting Information), the chemical composition of the DMCS includes C, N, S, and O. The atomic ratios of doped N and S in the DMCS are ≈1.92 and 0.04 at%, which are consistent with the EDS elemental maps. In the high‐resolution XPS spectrum of C 1*s* (Figure S2g, Supporting Information), four peaks at binding energies of 284.6, 285.5, 287.2, and 290.0 eV are indexed as C = C/C–C, C–N/C–S/C–O, C = O, and HO–C = O, respectively.^[^ ^29^ ^]^ The deconvolution of N 1 s from DMCS (Figure S2h, Supporting Information) illustrates the existence of pyridinic nitrogen (N‐6), pyrrolic nitrogen (N‐5), and graphitic nitrogen (N‐Q).^[^ ^30^ ^]^ It is widely known that N doping can render numerous electrochemically active sites for potassium‐ion storage and enhance the electronic conductivity of carbon substrate.[^8^, ^31^] The sulfur bonding was analyzed by fitting the high‐resolution S 2p spectrum (Figure S2i, Supporting Information) deconvoluted into two signals of C–S–C and S–O–S bonds, proving the successful doping of S into the carbon matrix, which can enhance the overall potassium storage capacity based on the increased reversible K^+^ binding sites.^[^ ^32^ ^]^

**Figure 3:**
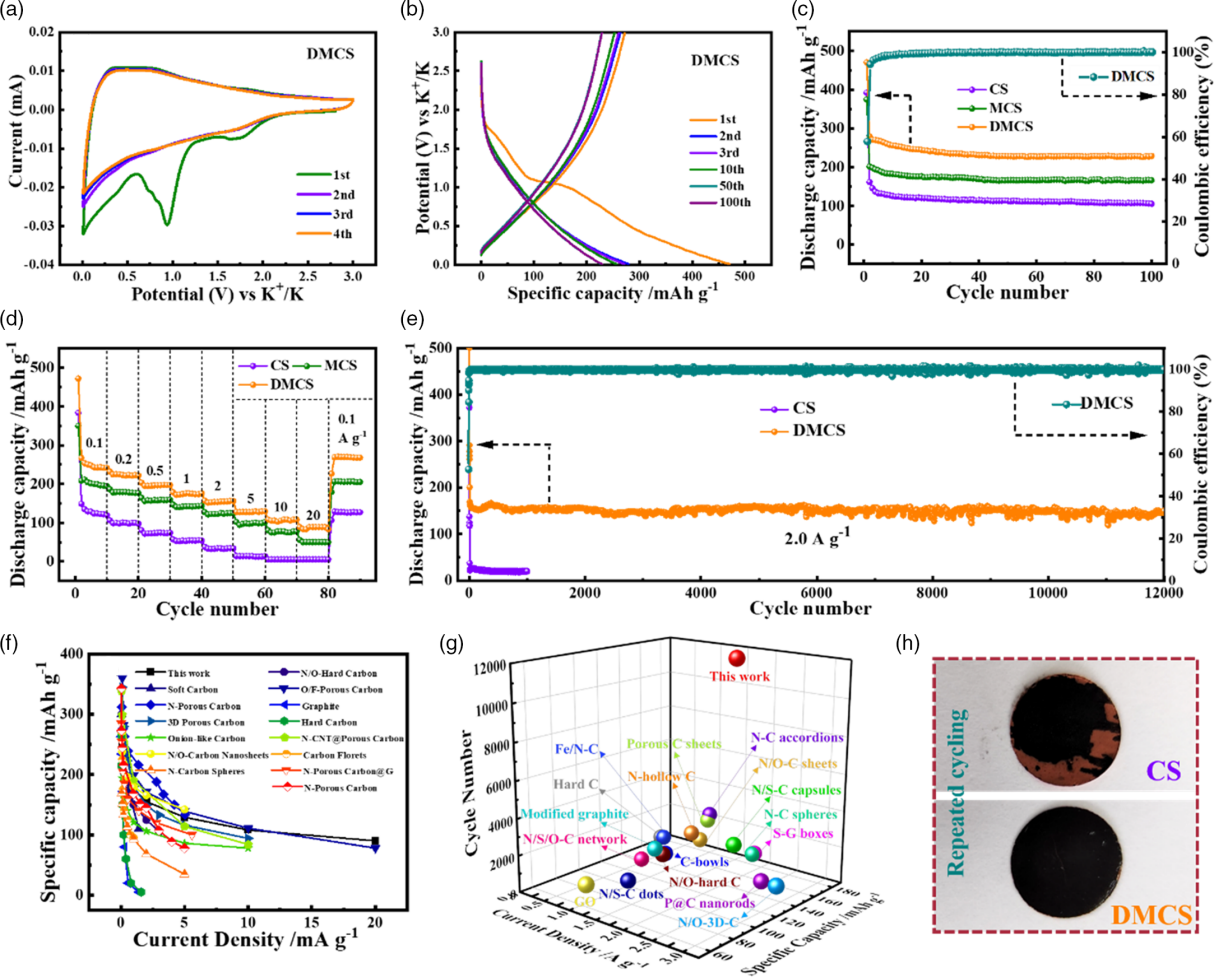
Electrochemical performances of DMCS as KIB anodes in half cells. a) CV curves at a scanning rate of 0.1 mV s^−1^. b) GCD profiles from the 1st to 100th cycles. c) Cycling performance at 0.1 A g^−1^. d) Rate capability at various current densities. e) Ultralong cycling performance at 2.0 A g^−1^. f,g) Comparison of rate properties (f ) and long‐term cycling performance (g) between DMCS and the other reported carbon‐based electrodes. h) Digital photographs of CS and DMCS electrodes after 100 cycles.

The electrochemical performance of the as‐prepared carbon‐based anodes was evaluated in half coin cells. **Figure** **3**a shows the first four cyclic voltammetry (CV) of the DMCS anode at a scan rate of 0.1 mV s^−1^ in the voltage window of 0.01–3.0 V. The initial discharge process of the CV curve exhibits a broad cathodic peak at 1.0 V due to the formation of irreversible solid electrolyte interphase (SEI) film on the electrode's surface.[^8^, ^33^] Subsequently, a sharp peak at the lower potential range of 0.01–0.3 V is observed, which can be ascribed to the insertion of K^+^ in the DMCS. During the following cycles from the second onward, there are no obvious changes in the area covered by CV curves, explicating the highly reversible K‐storage behavior of the DMCS electrode. Figure 3b presents the galvanostatic charge–discharge (GCD) voltage profiles for the 1st, 2nd, 3rd, 10th, 50th, and 100th cycles at a current density of 0.1 A g^−1^. The DMCS electrode delivers a first cycle discharge capacity of 470.5 mAh g^−1^ and a first cycle charge capacity of 272.5 mAh g^−1^, corresponding to a high initial Coulombic efficiency (ICE) of 57.9%. The irreversible capacity in the first charging process is mainly attributed to the formation of SEI film and the irreversible K^+^ insertion. Notably, the CE value of the DMCS anode increases rapidly to ≈95% during the second cycle, and then gradually stabilizes at >99% after 10 cycles, illustrating a good cycling stability. Most importantly, the curves of the 10th, 50th, and 100th cycles are nearly the same. Moreover, the typical sloping potassiation/depotassiation curve evidences the capacitive‐dominated K^+^ storage behavior. Figure 3c compares the cycling performance of the CS, MCS, and DMCS at 0.1 A g^−1^ for KIBs. After 100 cycles, the DMCS electrode achieves a reversible discharge‐specific capacity of 228.7 mAh g^−1^. The discharge‐specific capacities of MCS and CS electrodes are 151.7, and 105.5 mAh g^−1^, respectively. Meanwhile, MCS electrode show relatively higher capacity retention (≈82%) in comparison to that of CS (65%), which indicates that the channel architecture in the mesoporous carbon spheres is essential in optimizing the cycling stability of KIBs. Most importantly, the reversible capacity achieved by DMCS is obviously higher than that of MCS, which may be mainly attributed to the enhanced surface K‐ions adsorptive ability through the introduction of heteroatoms.

The rate capability and the cycling stability of batteries are critical for their practical applications, and the rate performance of the three anodes at different current densities are presented in Figure 3d and S3a, Supporting Information. Both MCS and DMCS exhibit higher rate capabilities compared with CS. The DMCS anode delivers discharge specific capacities of 267, 225, 197, 176, 153, 128, 108, and 89 mAh g^−1^ at current densities of 0.1, 0.2, 0.5, 1.0, 2.0, 5.0, 10.0, and 20.0 A g^−1^, respectively. In contrast, the corresponding discharge‐specific capacities for the MCS electrode are 209, 179, 159, 143, 123, 97, 78, and 50 mAh g^−1^, respectively. The rate capability of DMCS is comparable or superior to previously reported carbon‐based anodes for KIBs (Figure 3f).^[^ ^33^, ^34^ ^]^ Rate performance is generally controlled by electronic and ionic conductivity. The excellent rate capability of the DMCS is mainly related to the following aspects: i) the mesoporous structure with abundant radial pore channels is beneficial to the rapid ion diffusion and the stress‐buffering effect during the (de)potassiation process; ii) the doping of heteroatoms can provide plentiful defects/active sites for the adsorption and desorption of *K* ions, resulting in the improvement of the overall reversible specific capacity. As shown in Figure 3e, the long‐term cycling stabilities of the DMCS were characterized at a current density of 2 A g^−1^. Surprisingly, the DMCS anode achieves an impressively ultralong cycling life of over 12 000 cycles with a high capacity retention of ≈90% compared with the 7^th^ cycle. In detail, when the cell is cycled 12 000 times, the promising reversible specific discharge capacity maintains at 149.5 mAh g^−1^ with ≈100% Coulombic efficiency. It should be noted that there is no distinct decay in the whole cycle, which corresponds to an ultralow capacity decay rate of only 0.0082% per cycle. This ultralong cycling life of 12 000 cycles at 2 A g^−1^ outperforms other previous results of carbonaceous anode materials in KIBs (Figure 3g).^[^ ^8^, ^17^, ^33^, ^34^ ^h,^ ^35^ ^]^ In contrast, the CS suffers from fast reversible capacity fading of 20 mAh g^−1^ after 10 cycles, which can be associated with the structural damage of the CS electrode during cycling. Interestingly, even at a high current density of 5 A g^−1^, the DMCS anode delivers a reversible storage capacity of 97.2 mA h g^−1^after 6000 cycles (Figure S3b, Supporting Information). The exceptional long‐term cycling stability at 10 A g^−1^ can be obtained after over 3 500 cycles. Generally, electrolytes impose important effects on the electrochemical performance in energy storage systems.^[^ ^36^ ^]^ Despite the DMCS anode performing well in half coin cells, the highly concentrated DME‐based electrolytes may significantly increase costs in practical applications. Therefore, it is expected that further optimization of electrolytes should be achieved in the next work. Lu group reported a low concentrated 1,2‐diethoxyethane (DEE)‐based electrolyte enabling the graphite anode the superior electrochemical performance due to the weak K^+^‐DEE interactions and the solvating environment of K^+^.^[^ ^37^ ^]^ Inspired by this, the low concentration DEE‐based electrolyte may be conducive to the further enhancement of the electrochemical property of DMCS.

To understand the structural evolution of the electrode, a post‐mortem study was conducted subsequent to deep cycling. The CS and DMCS electrodes show the expected different macroscopic morphologies (Figure 3h). Specifically, the DMCS electrode maintains good integrity, while the CS electrode shows undesirable cracks and fragmented sections with cracks, where the part of the active material has dropped off from the current collector. Furthermore, the microstructure and morphology of the CS and DMCS electrodes before/after 100 cycles were investigated by SEM examination (Figure S4, Supporting Information). Remarkably, the electrical isolation of CS particles can be clearly observed after 100 cycles (Figure S4b, Supporting Information), which arises from the severe volume expansion and the correspondingly loosened particle‐to‐particle contacts. In contrast, the original textural properties of the DMCS electrode are well maintained upon repeated potassiation/depotassiation processes (Figure S4c,d, Supporting Information), proving the stress‐buffering effect of the mesoporous structure. The mesoporous architecture can effectively buffer the mechanical stress caused by volume variation during the insertion/extraction of K^+^, contributing to the superior electrode structural integrity and cycling stability of DMCS.

**Figure 4:**
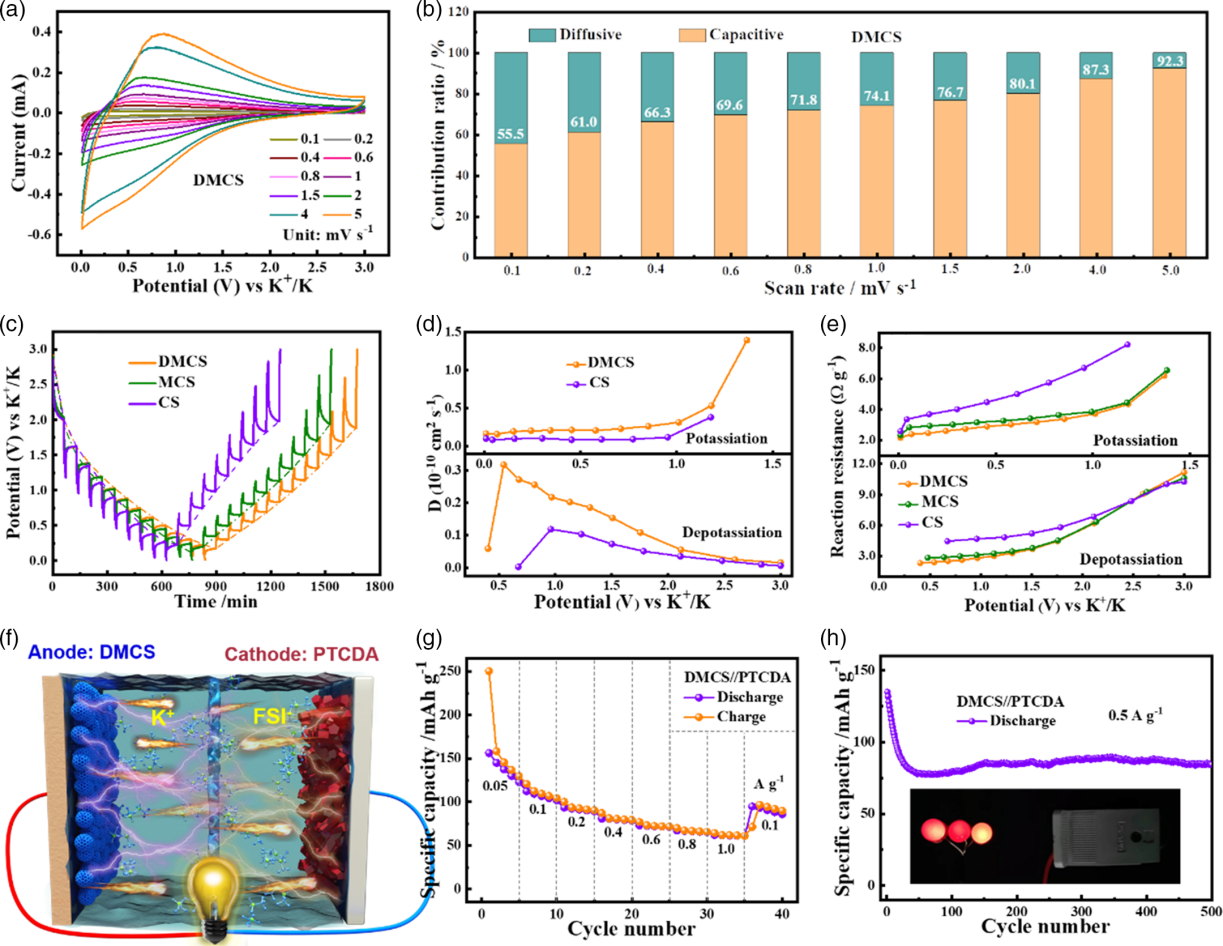
Analysis of K‐ion storage and diffusion kinetics. a) The cyclic voltammetry (CV) curves and b) capacitive contribution ratio of DMCS at different scan rates. c) galvanostatic intermittent titration technique (GITT) profiles, d) corresponding diffusion coefficients, and e) reaction resistances of these electrodes. f) Schematic illustration of the DMCS//perylene‐3,4,9,10‐tetracarboxylic dianhydride (PTCDA0‐450 K‐ion full battery. g) Rate capability and h) long cycling performance at 0.5 A g^−1^ of the full cell (based on active material of anode). The inset in (h) is the digital photograph of LEDs powered by our as‐assembled K‐ion full battery.

The kinetic analysis was investigated by conducting CV tests at different sweep rates ranging from 0.1 to 5.0 mV s^−1^ (**Figure** **4**a and S5a,b, Supporting Information). According to the equation *i*(*V*) = *k*_1_ *v* + *k*_2_ *v* ^1/2^, the current value (i) at a given potential (V) can be divided into the surface‐driven pseudocapacitive contribution (*k*_1_ *v*) and diffusion‐controlled contribution (*k*_2_ *v* ^1/2^).^[^ ^38^ ^]^ Figure S5c–e, Supporting Information, depicts the separations of the capacitive and diffusion currents of DMCS, CS, and MCS at a scan rate of 1.0 mV s^−1^. A dominant capacitive contribution of 66.1% at 1.0 mV s^−1^ is reached for MCS, which is significantly higher than 22.1% for CS, indicating that the mesoporous channel architecture can enhance the pseudocapacitive behavior. Additionally, the proportion of capacity contributed by the pseudocapacitive process increases with higher scan rates (Figure 4b and S5f,g, Supporting Information). Notably, the DMCS anode has the highest capacitive contribution at all scan rates. Even at a very low scan rate of 0.1 mV s^−1^, the DMCS anode shows a higher capacitive‐predominated contribution of 55.5%. Whereas, the CS anode is controlled by a diffusion behavior with an extremely low capacitive contribution of 8.2%. The simultaneous achievement of mesoporous architecture and surface defects introduced by heteroatom doping in the DMCS is responsible for the high fraction of pseudocapacitive contribution, which can improve the *K* ion transport rate and boost the rate capability and enhance the cycling life. To further elucidate the K^+^ diffusion kinetics during the dynamic potassiation/depotassiation process, the galvanostatic intermittent titration technique (GITT) test was carried out by applying a series of pulse current at 0.1 A g^−1^ for 10 min between rest intervals for 60 min (Figure 4c). Figure 4d shows the K‐ion diffusion coefficients of DMCS and CS calculated from GITT curves. Due to the advantage of the unique radial pore channels, DMCS shows a higher diffusion coefficient of K^+^ than CS in both charging and discharging processes, manifesting its better *K* ion diffusion kinetics. Significantly, the experimental analysis can correspond to the finding of theoretical calculations. The reaction resistances of three electrodes during (de)potassiation are calculated from GITT curves. As exhibited in Figure 4e, DMCS anode with N/S doping shows the minimum reaction resistance in comparison with CS and MCS during *K* intercalation/deintercalation processes. Electrochemical impedance spectroscopy (EIS) measurement was also conducted to explore the charge‐transfer properties. The Nyquist plots of these two electrodes after 20, 50, and 100 cycles are shown in Figure S6a, Supporting Information. The equivalent circuit is used to fit the EIS spectra (Figure S6b, Supporting Information), and the charge‐transfer resistances (*R*_ct_) values are presented in Figure S6c, Supporting Information. Furthermore, the DMCS electrode exhibits much lower *R*_ct_ than those of the CS anode, suggesting the good electrical conductivity and the low charge‐transfer resistance of DMCS. To evaluate the practical feasibility of DMCS, the K‐ion full cell was assembled by coupling with the previously reported organic cathode, perylene‐3,4,9,10‐tetracarboxylic dianhydride (PTCDA), as the cathode. To improve the electric conductivity of PTCDA, it was treated by an annealing process (450 °C for 4 h in argon atmosphere). As depicted in Figure S7, Supporting Information, the XRD pattern and morphology of PTCDA‐450 were measured and the corresponding electrochemical performance was studied in half‐cells with metallic potassium as a counter electrode. Figure 4f depicts the schematic illustration of the PTCDA‐450//DMCS K‐ion full cell. As shown in Figure S8a, Supporting Information, the full battery achieves a considerable discharge capacity of 156 mAh g^−1^ (based on anode mass) at 0.05 A g^−1^. The rate capability of PTCDA‐450//DMCS was further demonstrated (Figure 4g and S8b, Supporting Information), and the corresponding specific capacities are 110, 93, 81, 73, 67, and 62 mAh g^−1^ (based on anode mass) at different current densities of 0.1, 0.2, 0.4, 0.6, 0.8, and 1.0 A g^−1^, respectively. Impressively, the PTCDA‐450//DMCS presents good long‐term cycling stability up to 500 cycles with a specific capacity of 91 mAh g^−1^ at 0.5 A g^−1^ and a retention rate of 91% relative to the 5^th^ cycle (Figure 4h). As exhibited in the bottom inset of Figure 4h, the homemade full cell can easily illumine three light‐emitting diodes (LEDs).

**Figure 5:**
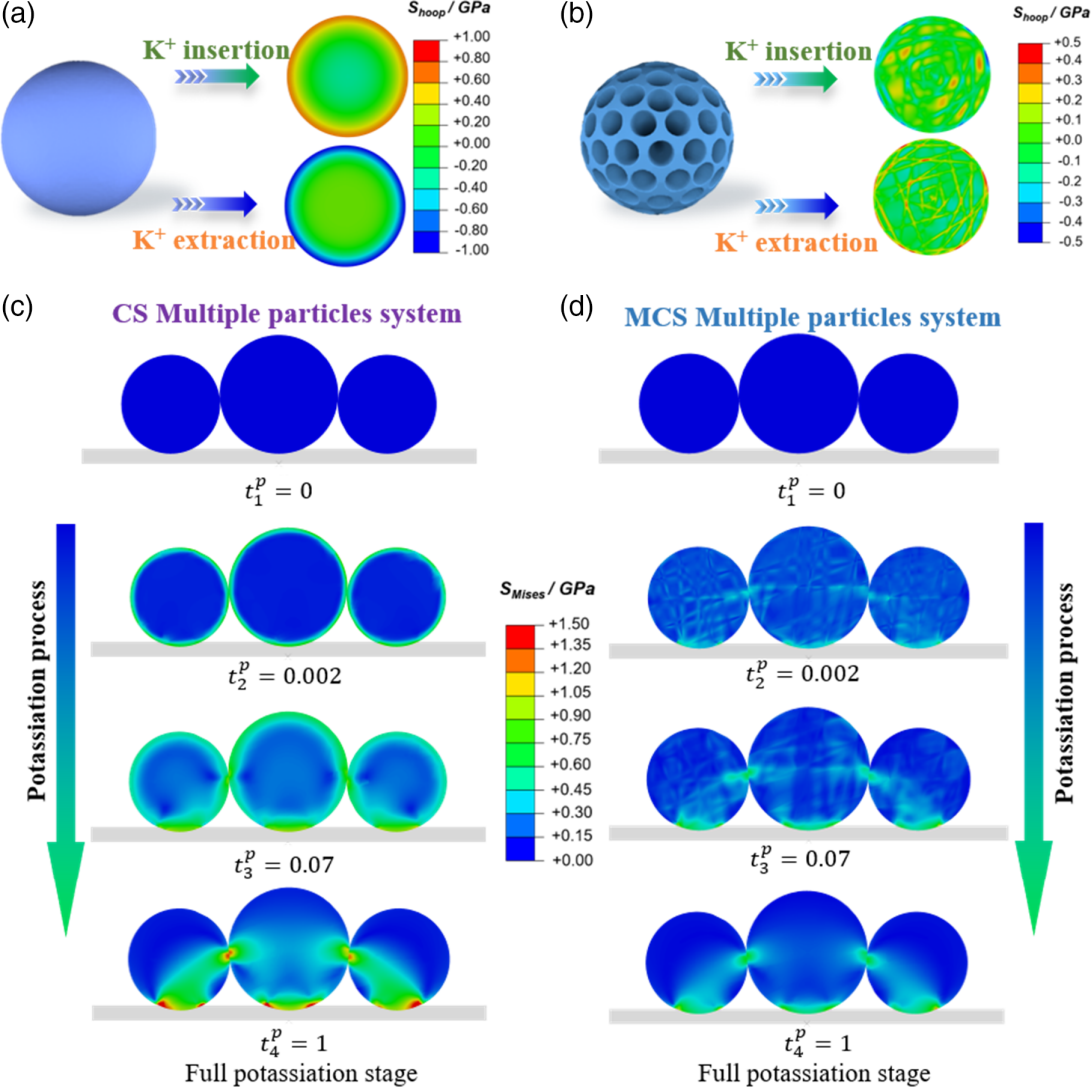
a,b) Cross‐sectional stress contours of single CS (a) and single MCS (b). c,d) Mises stress distribution in multiple CS (c) and multiple MCS (d) at various stages of potassiation. $\tilde{t}$ represents the normalized potassiation time, with $\tilde{t}$ = 0 representing the onset of potassiation and $\tilde{t}$ = 1 the time of full potassiation.

It is important to understand the mechanical stress evolution of carbon anodes during cycling since the potassiation‐induced stress is the main culprit of the mechanical degradation of electrodes. Herein, two mechanical models are built to study the stress profiles of the representative structures of individual carbon particles used in battery electrodes, including CS and MCS, respectively (Figure S9, Supporting Information). To focus on the effect of nanostructured configuration rather than that of chemical doping, we only compare the stress simulations of CS and MCS to decipher the origin of the outstanding long‐term cycling stability. Meanwhile, DMCS and MCS have similar morphology and internal microstructure. For the single‐particle CS system, the color contours shown in **Figure** **5**a and S10, S11, Supporting Information represent the magnitude of hoop stress. $\tilde{t}$ represents the normalized potassiation time, $\tilde{t}$ = 0 represents the onset of potassiation, and $\tilde{t}$ = 1 represents the time of full potassiation. The maximum stress value (i.e., 1.35 GPa) takes place on the outer surface of the CS particle (Figure S18, Supporting Information), which can cause the fracture of CS particles during the charge/discharge process. In contrast, in an MCS particle system, the regions with concentrated stress distribute near the surface of the channels in the form of sporadic points, with the maximum stress being 0.74 GPa (Figure 5b, S12, S13 and S18, Supporting Information), which is around only half of the maximum stress in a single CS particle. These results reveal the prominent stress‐alleviation effect of the MCS particle due to its mesoporous structure. Specifically, the MCS particle has a smaller Young's modulus than the CS particle according to the scaling law $Y\left(P\right)=E_{0}{\left(1-P\right)}^{3}$, where $E_{0}=32$ GPa is a constant and P=0.2 is the porosity.^[^ ^39^ ^]^ In addition, the mesoporous structure contains many channels for ion transport, which allow faster ion diffusion during the potassiation process. Hence, the ion distribution in the MCS particle is more uniform than that in the CS particle (Figure 5a,b), which greatly reduces the mechanical stress.

More significantly, we also investigated the stress evolution in a multiple‐particle system of CS and MCS, respectively, which is vital for understanding the mechanical failures in commercialized carbon‐based batteries. Figure 5c,d and S14–S17, Supporting Information, show the stress distribution of CS and MCS multi‐particle systems. For multiple CS particles, the high‐stress regions are still observed near the surface. In addition, stress concentration occurs at the contact regions among CS particles and the current collector during the insertion/extraction of K^+^. However, the MCS multi‐particle system undergoes drastically reduced stress. In particular, the maximum stress value of the MCS system is 1.29 GPa, which is smaller than that of the CS (2.73 GPa) (Figure S18, Supporting Information), revealing that the MCS electrodes are less vulnerable to the shedding and pulverization of active materials. The basic mechanism underpinning the stress‐mitigation effect in the MCS multi‐particle system lies in the diminished Young's modulus due to the mesoporous structure of MCS particles, which reduces the stress level at the contact regions and protects the electrodes from mechanical degradation. In summary, the stress‐buffering effect of the MCS anode via its mesoporous structure has been quantitatively uncovered by the finite element simulation.

## 3 Conclusions

We have developed a controllable self‐assembly strategy by the combination of the extended Stöber method and single‐micelle template approach to synthesize uniform dual‐heteroatom doped mesoporous carbon spheres with unique channel architecture from the molecule level. The obtained DMCS anode delivers outstanding rate capability (90 mAh g^−1^ at 20 A g^−1^) and ultrahigh cycling stability with almost no degradation (0.0082% decay rate with the remaining capacity of 149.48 mAh g^−1^) after >12 000 cycles at 2 A g^−1^, representing one of the best cycling stabilities. More importantly, we identify the linkages between nanoengineering design and ultrastable potassium‐ion storage from the perspective of stress regulation. Particularly, the finite element simulations coupling with experimental analyses reveal that the mesoporous structure plays a critical role in the stress‐buffering effect in the carbon‐based system, which can effectively prevent the stress concentration on the particles’ surface. The mesoporous structure is proved to be effective in reducing the stress through its interaction among particles and the current collector, releasing the mechanical stress upon cycling, thus avoiding the electrical isolation of nanoparticles and the delamination of the electrodes from the current collector, and eventually resulting in the exceptional rate capability and ultralong cycling life of the DMCS anode. This work provides a promising route for stress regulation with identified techniques for buffering mechanical stress originating from large volume fluctuations in advanced KIBs electrodes.

## Conflict of Interest

The authors declare no conflict of interest.

## Supporting information

Supplementary Material

## Data Availability

The data that support the findings of this study are available in the supplementary material of this article.
