# Supplementary material for: Stress‐Regulation Design of Mesoporous Carbon Spheres Anodes with Radial Pore Channels Toward Ultrastable Potassium‐Ion Batteries
Source: Small Sci. 2022 Sep 20;2(10):2200045. doi: 10.1002/smsc.202200045 (PMC11935954; doi:10.1002/smsc.202200045)
Supplement: Supplementary file 1 — Supplementary Material [file SMSC-2-2200045-s001.pdf]

Supporting Information

Stress-Regulation Design of Mesoporous Carbon Spheres Anodes with Radial Pore Channels towards Ultra-stable Potassium-ion Batteries

Shuming Dou, Qiang Tian, Tao Liu, Jie Xu, Lingyan Jing, Cuihua Zeng, Qunhui Yuan, Yunhua Xu, Zheng Jia, Qiong Cai, Wei-Di Liu, S. Ravi P. Silva, Yanan Chen*, and Jian Liu**

S. Dou, J. Xu, C. Zeng, [*] Prof. Y. Chen, Prof. Y. Xu
School of Materials Science and Engineering, Key Laboratory of Advanced Ceramics and Machining Technology of Ministry of Education
Tianjin Key Laboratory of Composite and Functional Materials
Tianjin University
Tianjin 300072, China
Email: yananchen@tju.edu.cn

Q. Tian, L. Jing, [*] Prof. J. Liu
State Key Laboratory of Catalysis, Dalian Institute of Chemical Physics, Chinese Academy of Sciences
Dalian National Laboratory for Clean Energy
Dalian 116023, China
E-mail: jianliu@dicp.ac.cn

T. Liu, [*] Prof. Z. Jia
Key Laboratory of Soft Machines and Smart Devices of Zhejiang Province, Center for X-Mechanics, Department of Engineering Mechanics
Zhejiang University
Hangzhou 310027, China
E-mail: zheng.jia@zju.edu.cn

S. Dou, Prof. Q. Quan,
School of Materials Science and Engineering
Harbin Institute of Technology (Shenzhen)
Shenzhen 518055, China

Prof. Q. Cai, Prof. S. Silva, [*] Prof. J. Liu
DICP-Surrey Joint Centre for Future Materials, Department of Chemical and Process Engineering
Advanced Technology Institute
University of Surrey

Guilford, Surrey GU2 7XH, UK
E-mail: jian.liu@surrey.ac.uk

Dr. W. Liu, Prof. J. Liu
Australian Institute for Bioengineering and Nanotechnology
the University of Queensland
St Lucia, Brisbane, Queensland 4072, Australia

1. Experimental Section

1.1. Chemicals and Materials

Triblock poly(ethylene oxide)-b-poly(propylene oxide)-b-poly(ethylene oxide) pluronic F127 (Mw =12,600) was purchased from Sigma-Aldrich. Resorcinol ($C_6H_6O_2$), 3-aminophenol (C_6H_7NO), ammonium hydroxide (NH_4OH, 28~30wt%), formaldehyde solution (CH_2O, 37 wt %), trimethylbenzene (TMB, C_9H_{12}), and ethanol (C_2H_6O) were purchased from Shanghai Chemical. Resorcinol sulfide ($C_{12}H_{10}O_4S$) was purchased from Shanghai Macklin Biochemical Co., Ltd. All chemicals were of analytical grade and used without further purification. Deionized water was used for all experiments.

1.2. Material Synthesis

1.2.1. Modified Stöber Method to Prepare Carbon Spheres (CS)

In a typical synthesis, 1 mL of ammonia was added in a mixture of 15 mL of water and 15 mL of ethanol. Then, 0.4 g of resorcinol and 0.56 mL of formaldehyde was uniformly dispersed in the reaction mixture, followed by stirring for 12 h. The orange suspension was transferred to a sealed Teflon-lined autoclave for aging at 100 ºC for 24 h. Resorcinol-formaldehyde spheres (RFS) were collected by centrifugation and drying. Carbonization of RFS was carried out under a N_2 flow. The tubular furnace was heated

at a heating rate of 5 °C min^{-1} and maintained at 350 °C for 2 h. Afterward, the temperature was raised to 800 °C at a rate of 5 °C min^{-1} and maintained for 5 h. Finally, the CS powder was obtained.

1.2.2. Preparation of MCS

In a typical synthesis, 0.13 g of block copolymer F127 was dispersed in a mixture of 15 mL of water and 15 mL of ethanol. 0.4 mL of TMB and 1.0 mL of ammonia were added to the reaction mixture. Once the mixture became milky white, 0.56 mL of formaldehyde and 0.4 g of resorcinol were added, followed by stirring for 12 h. The orange suspension was transferred to a sealed Teflon-lined autoclave for aging at 100 ºC for 24 h. Mesoporous resorcinol-formaldehyde spheres (MRFS) were collected were collected by centrifugation and drying. Carbonization of MRFS was carried out in a flow of N_2 gas. The tubular furnace was heated at a heating rate of 5 °C min^{-1} and maintained at 350 °C for 2 h. Afterward, the temperature was raised to 800 °C at a rate of 5 °C min^{-1} and maintained for 5 h. Finally, the MCS powder was obtained.

1.2.3. Preparation of DMCS

DMCS was prepared using the same method as MCS, except that resorcinol (0.4 g) is substituted with the mixture of 3-aminophenol (0.2 g) and resorcinol sulfide (0.2 g).

1.3 Materials Characterization

HT7700 (Japan) was used for transmission electron microscopy (TEM) at 100 kV. Hitachi-S5500 (Japan) was used to obtain scanning electron microscopy (SEM) images. On a Rigaku D/Max2500PC diffractometer, powder X-ray diffraction data were

collected over the 2θ range of 5°-80° with a scan speed of 5 ° min^{-1} at room temperature using Cu K* radiation (θ=1.5418). Micromeritcs ASAP 2460 and Tristar 3020 were used to measure the N_2 sorption isotherms at 77 K. Before measurement, the samples were placed in a vacuum at 200 °C for at last 8 h. Photoelectron spectroscopy (XPS) was performed using a monochromatic Al source on KRATOS, Axis Ultra. All of the binding energies were modified using the C 1s standard peak (284.6 eV) was used to revise all binding energies. The spectrometer (NanoWizard Ultra Speed &inVia Raman) was used to obtain Raman data with an excitation wavelength of 532 nm.

1.4 Electrochemical Characterization

The working electrode slurry was a mixture of the carbon-based materials, Super P and sodium alginate (SA) with a weight ratio of 80:10:10. The uniform black slurry was pasted onto Cu foil and vacuum-dried at 60 ºC for 12 h. The average mass loading of each electrode was about 0.8-1.0 mg cm^{-2}. The electrolyte is 3M KFSI dissolved in DME. A glass fiber filter (Whatman GF/D) was applied as the separator, and K metal was used as the counter electrodes. Neware battery testing system was employed to conduct Galvanostatic charge-discharge (GCD). CHI660E electrochemistry workstation and AUTOLAB electrochemistry workstation were used to measure cyclic voltammetry (CV) and electrochemical impedance spectroscopy (EIS).

1.5 Finite Element Modelling

The thermal diffusion and expansion model in commercial FEM package ABAQUS was used to simulate the volume-expansion of carbon particles, because the governing equations for mass diffusion and associated volume change take the same

form as those for thermal diffusion and expansion. In the modeling, the MCS is modeled as an elastic material. Young's modulus of mesoporous carbon is estimated by $Y(P) = E_0(1-P)^3$, where E_0 = 32 GPa, is the Young's modulus of solid carbon, and P is the porosity. For $P \approx 0.2$, Young's modulus is approximately 16.4 GPa. And the volume expansion induced by ion intercalation is modeled via a thermal-expansion analogy, such that the linear swelling strain is given by $\alpha_v \Delta T$, where $\alpha_v = 0.1$, is the thermal expansion coefficient and ΔT $(0 \leq \Delta T \leq 1)$ the temperature change set in the simulation. We assign a temperature change of $\Delta T = 1$ to both the outer surface as well as the percolating channels inside the MCS particle, to model the ion diffusion and volume expansion during the charging process.

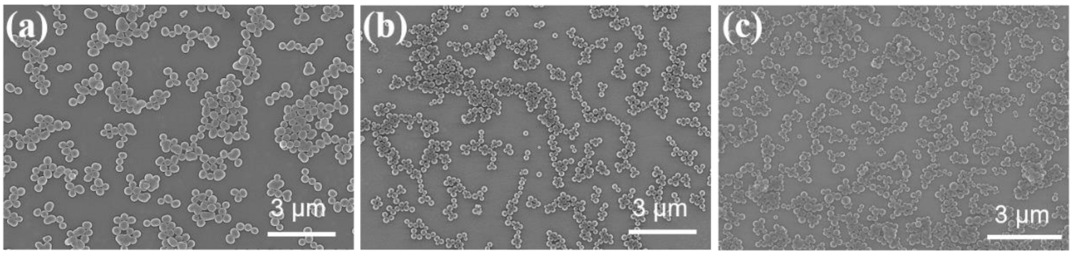

Figure S1. Low-magnification SEM images of (a) CS, (b) MCS and (c) DMCS.

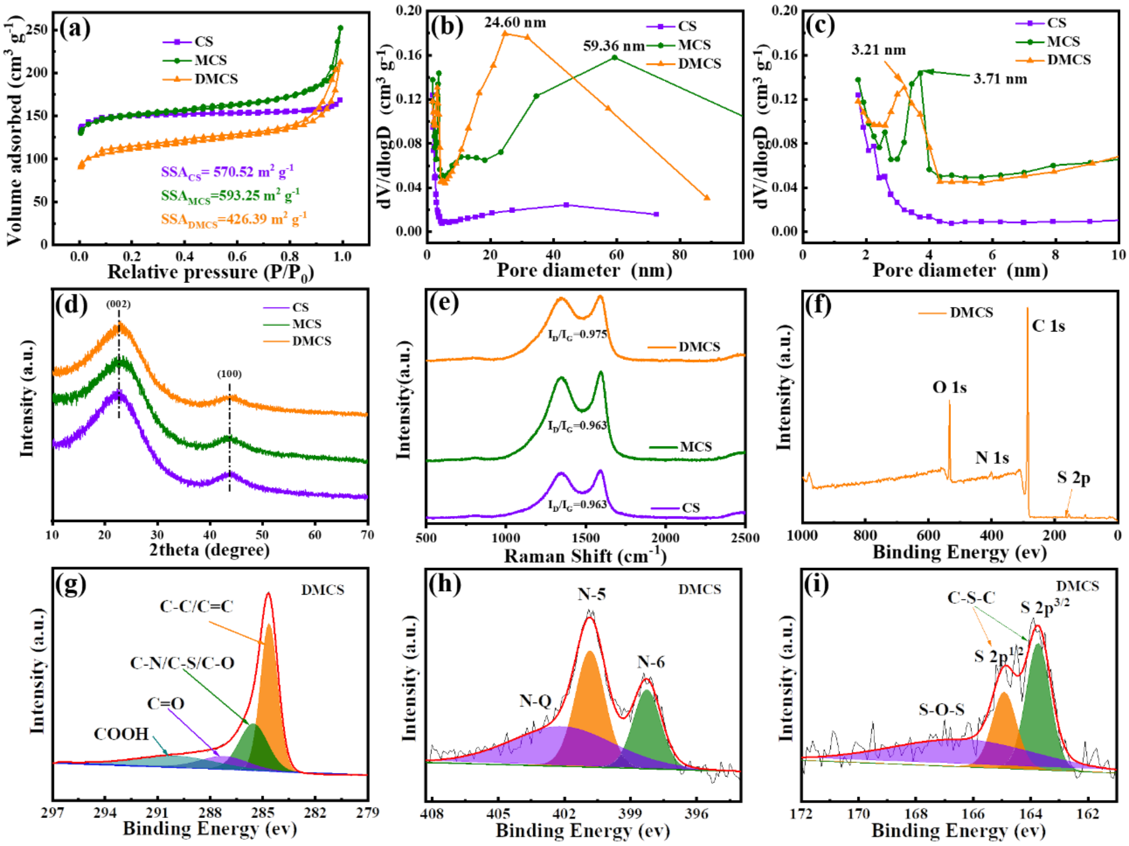

Figure S2. (a) N_2 adsorption-desorption isotherm curves and (b, c) Pore-size distribution curves of CS, MCS and DMCS. (d) XRD patterns and (e) Raman spectra of CS, MCS and DMCS. (f) XPS survey spectrum of DMCS. High-resolution XPS spectra of (g) C 1s, (h) N 1s, and (i) S 2p for DMCS.

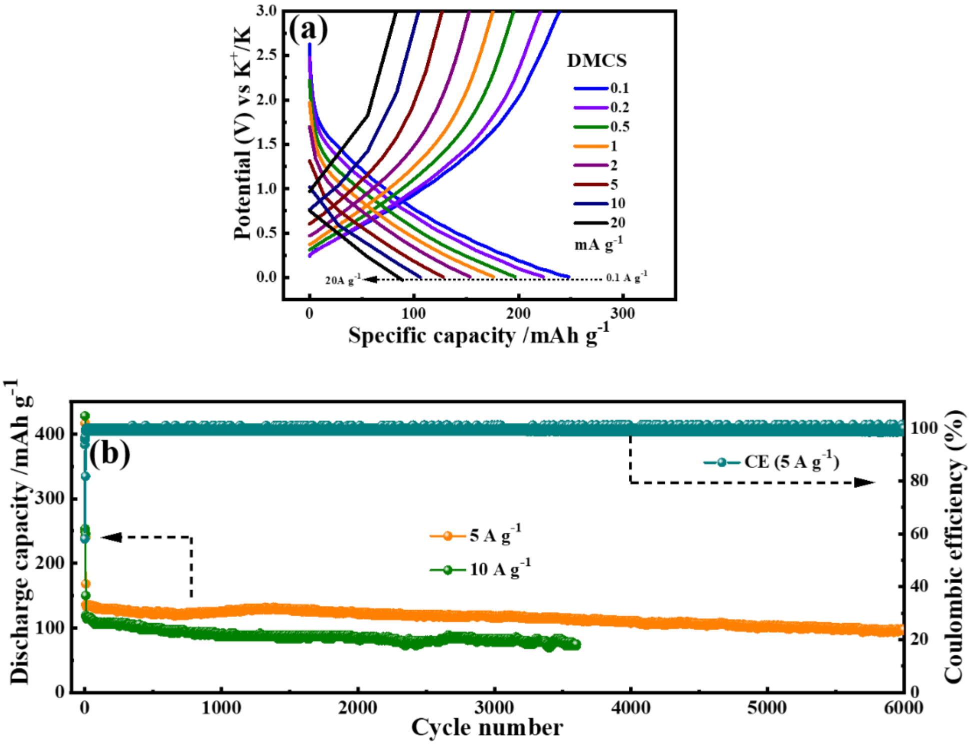

Figure S3. (a) Charge-discharge profiles of NS-MCS at various current densities. (b) Long-term cycling performances of NS-MCS at a high current density of 5 and 10 A g^{-1}.

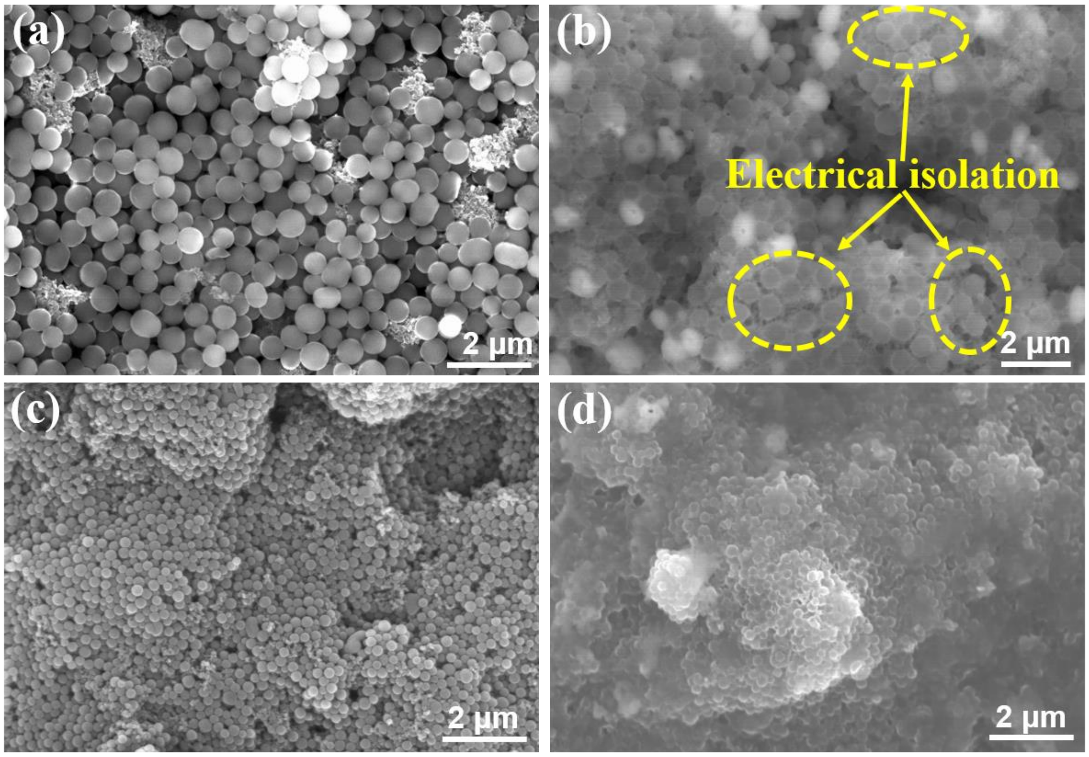

Figure S4. SEM images of (a) CS and (c) DMCS before cycling. SEM images of (b) CS and (d) DMCS after 100 cycles.

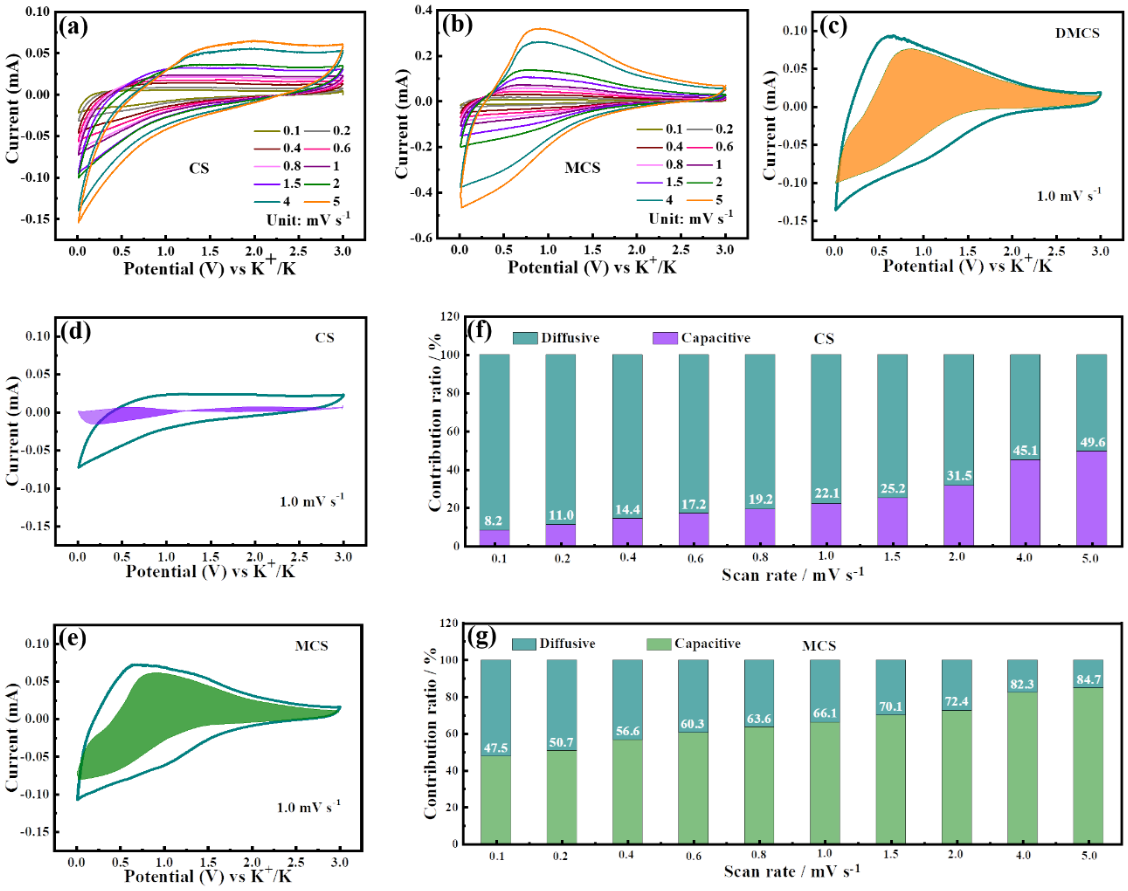

Figure S5. CV curves of (a) CS and (b) MCS at various scan rates. Separations of the capacitive and diffusion currents of (c) DMCS, (d) CS and (e) MCS at a scan rate of 1.0 mV s^{-1}. Capacitive contribution ratios of (f) CS and (g) MCS at different scan rates.

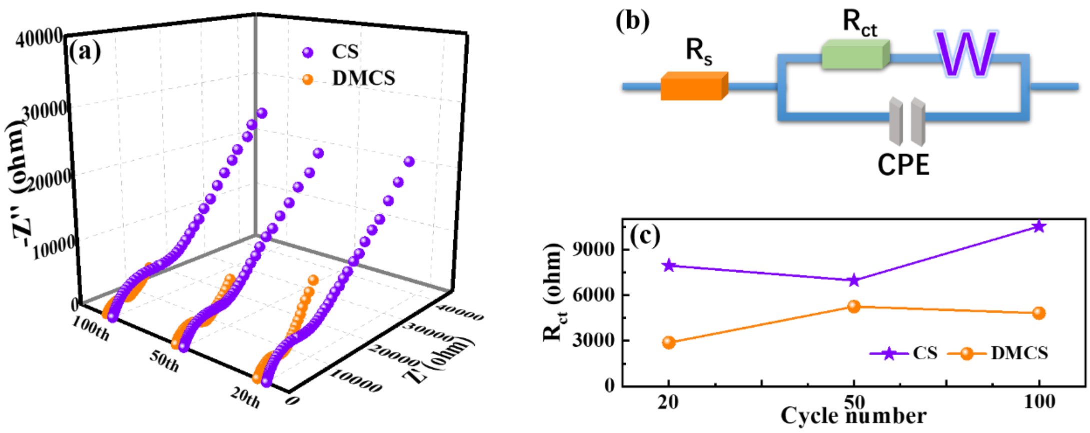

Figure S6. (a) EIS of CS and DMCS electrodes after 20, 50 and 100 cycles. (b) The corresponding equivalent circuit used to simulate EIS curves. (c) R_{ct} of CS and DMCS anodes derived from the equivalent circuit.

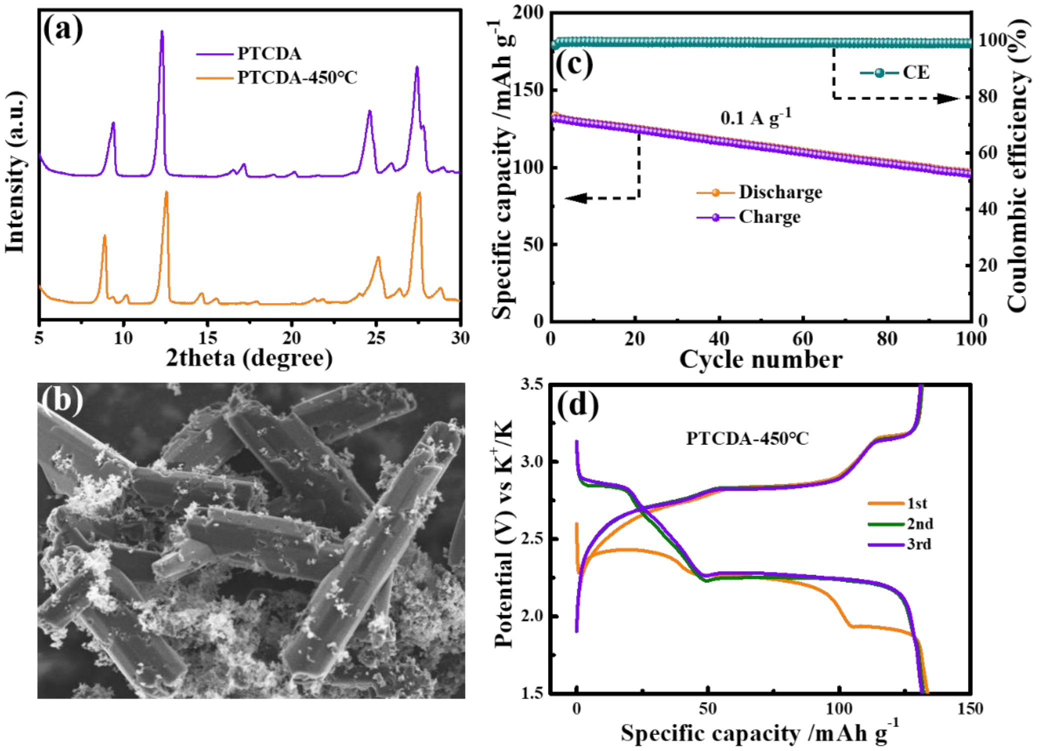

Figure S7. Microstructural and electrochemical evaluations of PTCDA-450 cathode material. (a) XRD patterns. (b) SEM image. (c) Cycling performance. (d) GCD profiles.

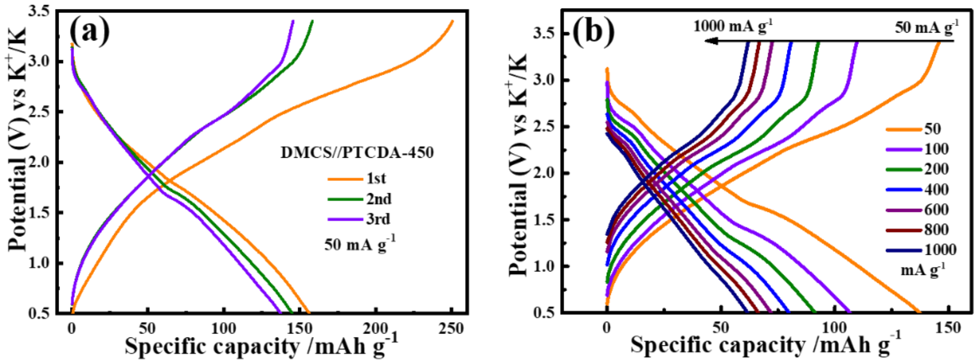

Figure S8. GCD profiles of the DMCS//PTCDA-450 K-ion full battery.

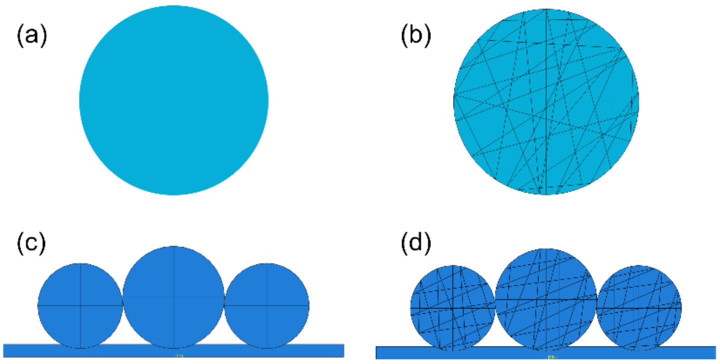

Figure S9. Cross-section views of 3D models of (a) single-particle CS, (b) single-particle MCS, (c) multiple-particle CS and (d) multiple-particle MCS. In (b) and (d), the solid lines inside the particles represent the channels in the porous anodes.

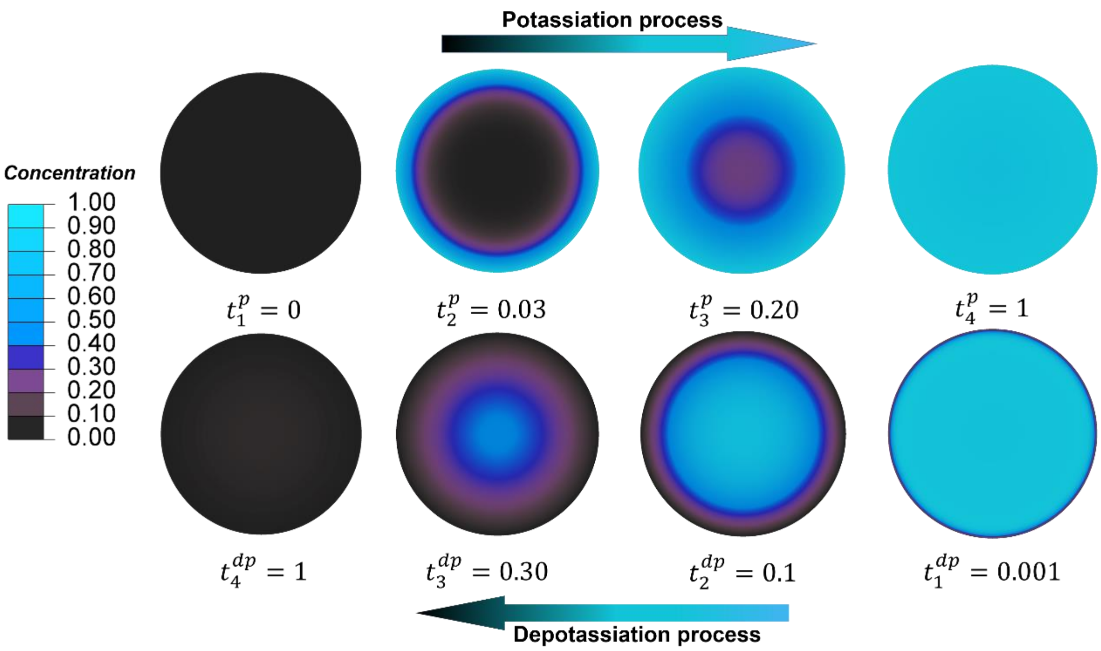

Figure S10. Chemomechanical modelling of the potassiation/depotassiation processes of single-particle CS. Colors denote the K concentration, with blue being fully potassiated and black unpotassiated.

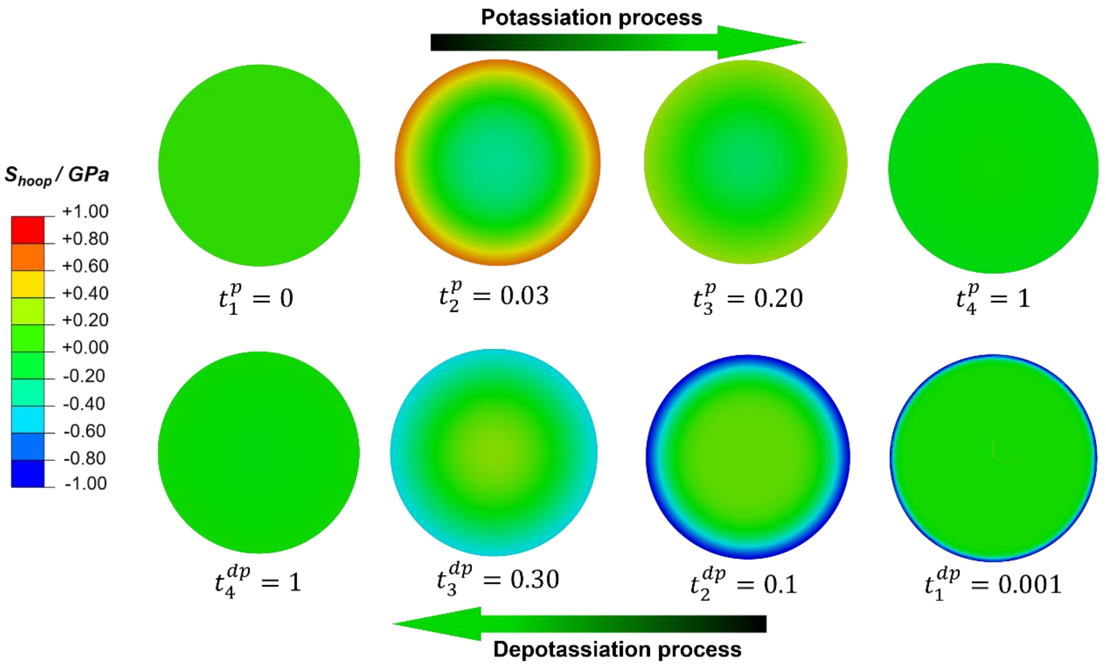

Figure S11. Hoop stress distribution in single-particle CS at various stages of potassiation/depotassiation. Positive means the tensile stress (red) while negative represents the compressive stress (blue).

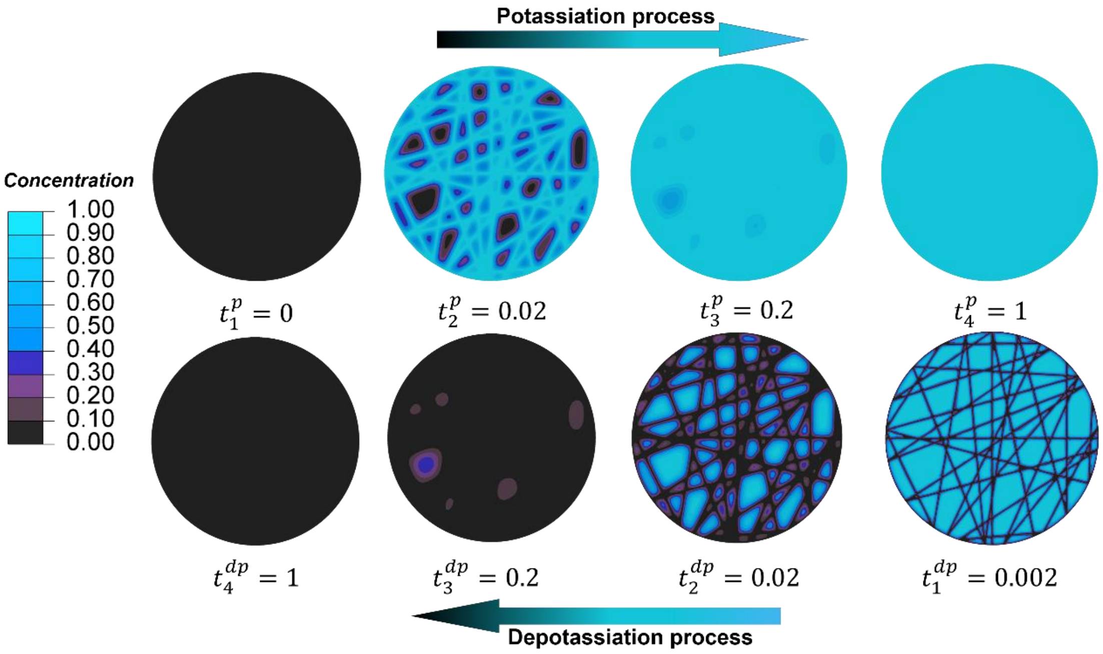

Figure S12. Chemomechanical modelling of the potassiation/depotassiation processes of single-particle MCS. Colours denote the K concentration, with blue being fully potassiated and black unpotassiated.

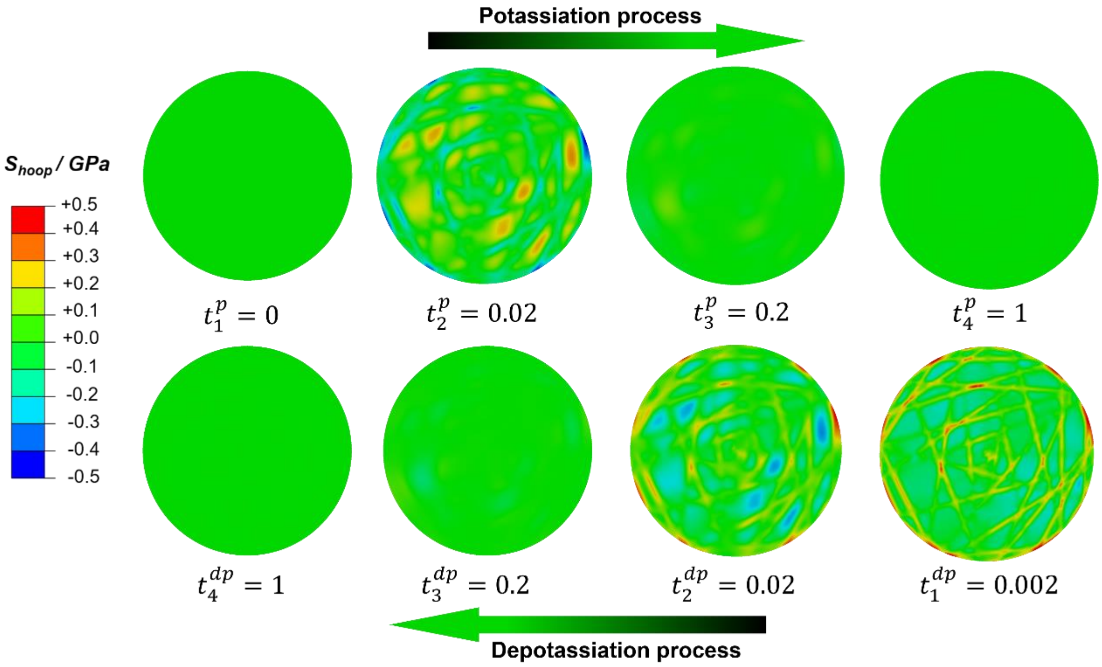

Figure S13. Hoop stress distribution in single-particle MCS at various stages of potassiation/depotassiation. Positive means the tensile stress (red) while negative represent the compressive stress (blue).

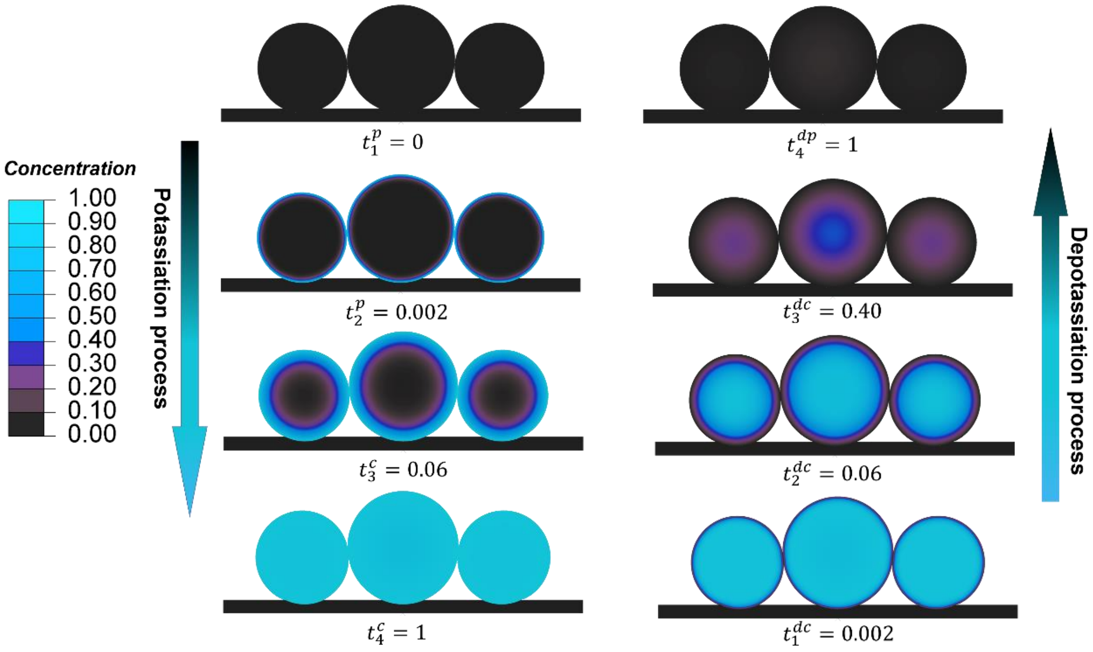

Figure S14. Chemomechanical modelling of the potassiation/depotassiation processes of multiple-particle CS. Colors denote the K concentration, with blue being fully potassiated and black unpotassiated.

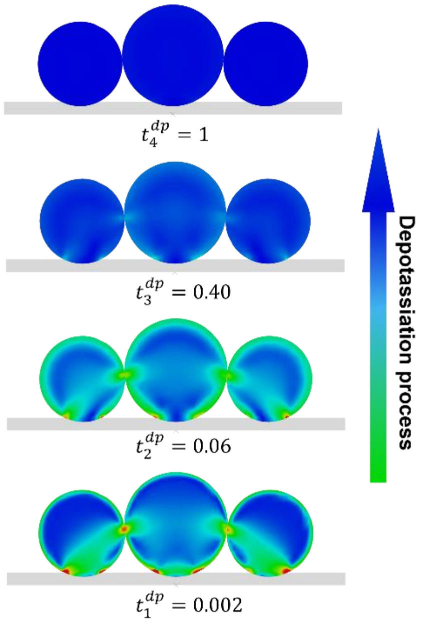

Figure S15. Mises stress distribution in multiple-particle CS at various stages of depotassiation. Positive means the tensile stress (red) while negative represent the compressive stress (blue).

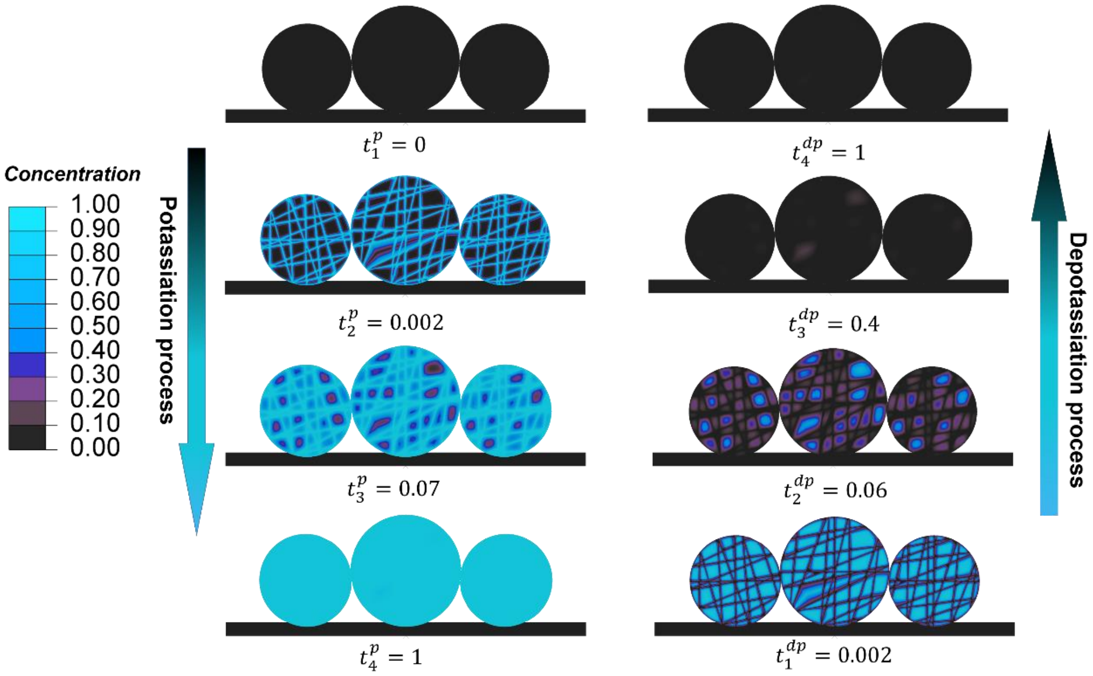

Figure S16. Chemomechanical modelling of the potassiation/depotassiation processes of multiple-particle MCS. Colors denote the K concentration, with blue being fully potassiated and black unpotassiated.

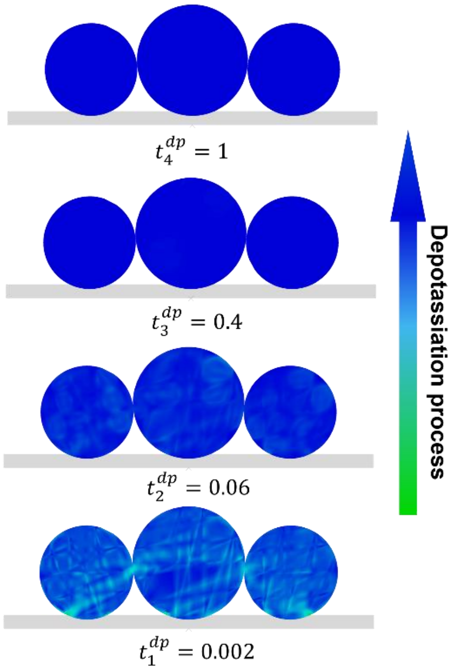

Figure S17. Mises stress distribution in multiple-particle MCS at various stages of depotassiation. Positive means the tensile stress (red) while negative represent the compressive stress (blue).

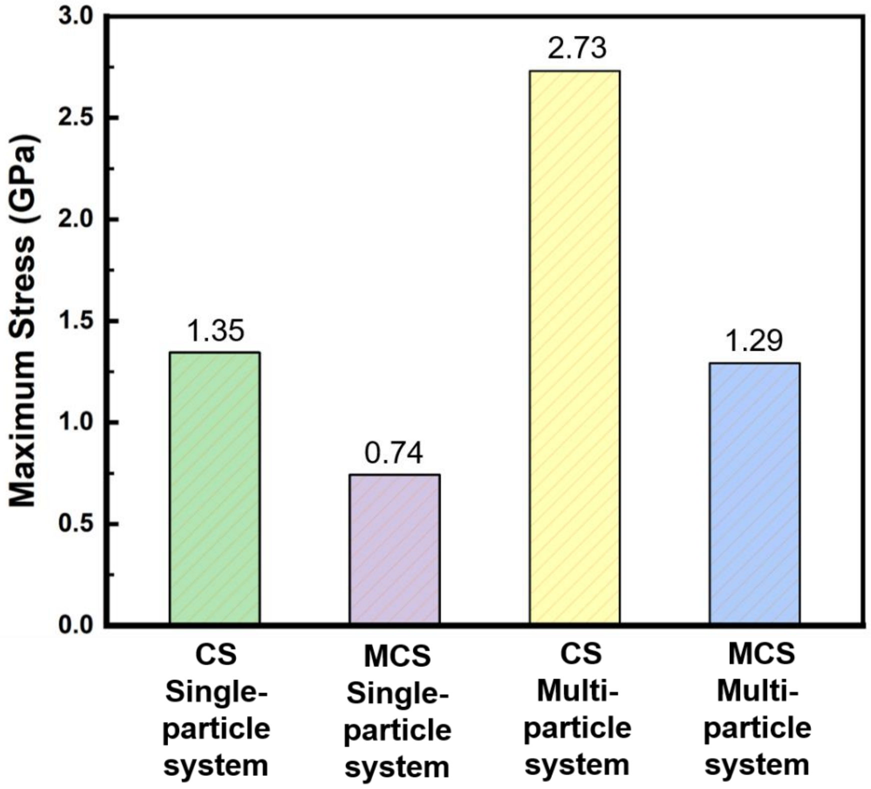

Figure S18. Comparison of maximum stress of CS and MCS single-/multi-particle systems.
